# Intraoperative Pancreatic Ductoscopy for Ampullary Adenocarcinoma During Pancreatic Resection: A Case Report

**DOI:** 10.1089/pancan.2019.0005

**Published:** 2019-10-10

**Authors:** Anthony Congiusta, Ariel Brown, Andrew M. Brown, Charles J. Yeo

**Affiliations:** Department of Surgery, Jefferson Pancreas, Biliary, and Related Cancer Center, Thomas Jefferson University, Philadelphia, Pennsylvania.

**Keywords:** periampullary neoplasm, intraoperative pancreatic ductoscopy, pylorus-preserving pancreaticoduodenectomy

## Abstract

**Background:** Periampullary neoplasms can be challenging to work up and diagnose preoperatively. Herein, we report the case of a patient whose preoperative workup failed to detect a malignancy, yet, underwent a pylorus-preserving pancreaticoduodenectomy (PPPD) with intraoperative pancreatic ductoscopy (IPD) and was ultimately found to have an ampullary adenocarcinoma.

**Presentation:** A 78-year-old woman presented with 4 weeks of nausea, weight loss, jaundice, and light-colored stools. She underwent outpatient diagnostic studies, including magnetic resonance cholangiopancreatography, endoscopic ultrasound, and endoscopic retrograde cholangiopancreatography with pancreatic duct (PD) stenting and papillotomy. These revealed common bile duct dilatation measuring 2 cm, PD dilatation measuring 7 mm, a 17 mm cyst in the head of the pancreas, and a firm nodule noted between the biliary and pancreatic orifices. Cytologic and pathologic analyses were initially nondiagnostic. A repeat ampullary biopsy was negative for dysplasia and malignancy. A computed tomography scan was then performed and showed cystic pancreatic lesions with pancreatic ductal dilation. Suspicion remained high for periampullary tumor or a main duct intraductal papillary mucinous neoplasm, and the patient underwent a PPPD with IPD and tolerated the procedure well. Her final specimen pathology revealed well-to-moderately differentiated ampullary adenocarcinoma, pancreaticobiliary type with positive nodal disease.

**Conclusions:** Given the relatively poor prognosis of patients with node-positive pancreaticobiliary-type ampullary adenocarcinoma, clinical suspicion should remain high for malignancy in patients with lesions located in the periampullary region and a negative preoperative workup, as aggressive treatment approaches are warranted to maximize their chance for survival.

## Introduction

Periampullary tumors are neoplasms within or around the ampulla of Vater and include pancreatic, bile duct, ampullary, and duodenal primaries. Working up the various types of periampullary tumors, and making an accurate preoperative diagnosis, can be challenging.^1,2^ Intraoperative pancreatic ductoscopy (IPD) has been reported to have greater sensitivity and specificity in detecting surgical pathology when compared to endoscopic retrograde cholangiopancreatography (ERCP) and endoscopic ultrasound (EUS). IPD has been shown to be safe and effective in evaluating main duct intraductal papillary mucinous neoplasms (MD-IPMN), with a specific advantage of diagnosing multicentric lesions.^3^

Herein, we report the case of a patient whose preoperative workup failed to detect ampullary malignancy, yet, underwent pylorus-preserving pancreaticoduodenectomy (PPPD) with IPD and was subsequently diagnosed with ampullary cancer.

## Case Report

A 78-year-old woman presented without abdominal pain, but with nausea, 10 pound weight loss, light-colored stools, and jaundice. She had an elevated alkaline phosphatase (372 U/L; normal <147 U/L) and mild transaminitis (aspartate aminotransferase 49 U/L, alanine aminotransferase 77 U/L; normal <40 and <56 U/L, respectively). Bilirubin levels were within normal limits (total 0.3 mg/dL; normal 0.1–0.9 mg/dL, and direct <0.2 mg/dL; normal <0.3 mg/dL), as were tumor markers at presentation (CA-19.9 13 U/mL; normal <35 U/mL, and carcinoembryonic antigen 1.8 ng/mL; normal <4.7 ng/mL). Magnetic resonance cholangiopancreatography initially noted dilation of the common bile duct to 2 cm and main pancreatic duct (PD) to 7 mm and ERCP were subsequently performed on the same day.

**FIG. 1.**
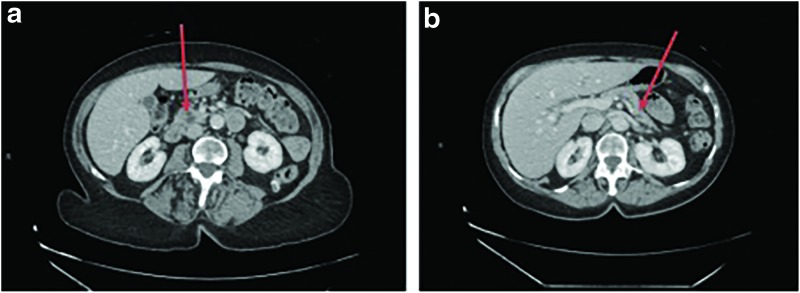
CT scan showing two cystic pancreatic lesions. Left panel **(a)** shows a 6 mm lesion in uncinate process (arrow). Right panel **(b)** shows a 5 mm lesion in tail of pancreas (arrow), and a dilated main pancreatic duct in the body and tail. CT, computed tomography.

EUS revealed a 17 mm head of pancreas (HOP) cyst communicating with the main PD, which was needle aspirated and drained. She then underwent ERCP with PD stenting done for clot noted in a very dilated PD. A papillotomy was performed with a firm nodule noted between the biliary and pancreatic orifices. Cytology and pathology from the HOP cyst and papillotomy specimen, respectively, were reported as nondiagnostic. A repeat endoscopic biopsy was negative for dysplasia and malignancy. A computed tomography scan revealed cystic pancreatic lesions within the uncinate process and tail measuring 6 and 5 mm, respectively (Fig. 1a, b), as well as noting that the PD was dilated to 9 mm.

Although initial workup failed to reveal malignancy, a high clinical suspicion remained for a periampullary neoplasm or main duct intraductal papillary mucinous neoplasm (MD-IPMN). IPMN was considered given the findings of proteinaceous, amorphous material in the fine needle aspiration specimen, suspicious for mucin. Benign epithelial cells were seen in the ampullary biopsy. The patient elected to undergo a PPPD. An exploratory laparotomy revealed a small palpable ampullary mass, worrisome for malignancy, but no evidence of metastasis. A cholecystectomy was performed, followed by kocherization of the duodenum and extirpation of the PPPD specimen. An IPD was performed on the pancreatic remnant using a 2 mm choledochoscope by inserting a flexible pancreatoscope into the transected main duct's proximal end and advancing it distally toward the pancreatic tail, but never reaching the tail due to the narrowing lumen of the main duct. The findings of the pancreatoscope were visualized by the surgical team on a monitor in real-time. The authors observed a dilated but otherwise normal PD without evidence of an IPMN, that is, neither papillary projections nor cobblestoning. Intraoperative histological assessment was performed which revealed an adenocarcinoma in the periampullary region with all margins reported as negative. Reconstruction with end-to-side pancreatico-, hepatico-, and duodeno-jejunostomies was performed.

**FIG. 2.**
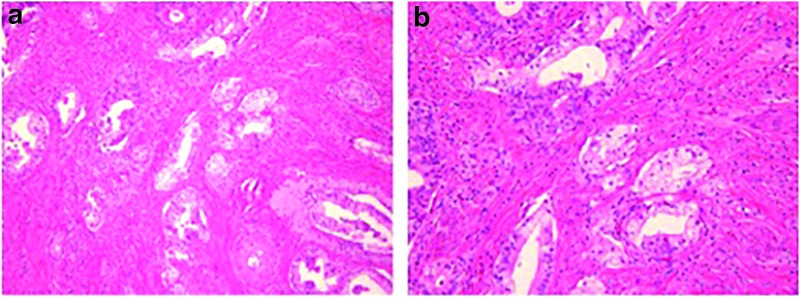
Final histopathologic slides. Histology of specimen (H&E stain): pancreaticobiliary subtype ampullary carcinoma at 10 × on the left **(a)** and 20 × on the right **(b)**. H&E, hematoxylin and eosin.

The patient tolerated the procedure well and her postoperative course was uncomplicated. She was discharged on postoperative day 5, as part of the Whipple Accelerated Recovery Pathway.^4^ Final pathology showed well-to-moderately differentiated ampullary adenocarcinoma, pancreaticobiliary type, with negative margins and 3 of 13 lymph nodes positive for metastatic disease (Fig. 2a, b). The patient will be consulting with a medical oncologist and a radiation therapist to discuss postoperative adjuvant therapy.

## Discussion

Primary ampullary tumors arise within the ampulla of Vater, making up 0.5% of all gastrointestinal malignancies.^5^ They account for ∼7% of periampullary cancers and typically present early in their disease course with symptoms of biliary obstruction such as jaundice.^2^ Patients may also have nonspecific symptoms, including diarrhea, weight loss, and fatigue. Histologic subtypes include intestinal (47%), pancreaticobiliary (24%), poorly differentiated adenocarcinoma (13%), intestinal-mucinous (8%), and invasive papillary (5%).^6^

The pancreaticobiliary subtype of ampullary adenocarcinoma is associated with the worst overall survival and disease-free survival.^7^ Surgical resection remains the only potentially curative treatment for ampullary carcinoma. Preoperative imaging and endoscopic techniques cannot always characterize periampullary lesions and standard intraoperative inspection and palpation may not provide enough information. IPD has been shown to influence real-time decision making, particularly when considering further pancreatic resection. It can also help rule out synchronous lesions in the pancreatic remnant, ductal neoplastic changes, and allow for direct visual inspection of the duct and any associated lesions.^8,9^

This case report illustrates an example of how IPD is used at a single institution to help clarify preoperative uncertainty and provide additional information regarding the decision of further pancreatic resection. In this case, due to a nondiagnostic workup, IPD was used to help rule out other ductal pathology, which, if present, may have necessitated further resection of the pancreatic remnant. The authors assert that IPD is simple to use and interpret and should be considered a valuable adjunct in the pancreatic surgeon's arsenal. At our institution, we perform over 200 pancreatic resections per year, and we utilize IPD, on average, once or twice per month.

## Conclusion

There is scant literature detailing the use of IPD to help differentiate ampullary adenocarcinoma from other ductal pathology. Despite negative preoperative workup, histopathologic analysis determined that the patient harbored an ampullary adenocarcinoma with node-positive disease. IPD was used in this case to rule out other pathologies in the pancreatic remnant. There was no evidence of papillary projections or cobblestoning, which would have been atypical for an ampullary adenocarcinoma. Given the poor prognosis of the lesion, aggressive treatment approaches are warranted to maximize chance for survival.

## Abbreviations Used

CT: computed tomography
ERCP: endoscopic retrograde cholangiopancreatography
EUS: endoscopic ultrasound
H&E: hematoxylin and eosin
IPD: intraoperative pancreatic ductoscopy
IPMN: intraductal papillary mucinous neoplasm
MD: main duct
PD: pancreatic duct
PPPD: pylorus-preserving pancreaticoduodenectomy
